# Planning date nights that promote closeness: The roles of relationship goals and self-expansion

**DOI:** 10.1177/02654075211000436

**Published:** 2021-03-17

**Authors:** Cheryl Harasymchuk, Deanna L. Walker, Amy Muise, Emily A. Impett

**Affiliations:** 1 6339 Carleton University, Canada; 2 6221 Western University, Canada; 3 7991 York University, Canada; 4 7938 University of Toronto Mississauga, Canada

**Keywords:** Approach relationship goals, date nights, intimate relationships, self-expansion model, shared leisure

## Abstract

Spending time with a romantic partner by going on dates is important for promoting closeness in established relationships; however, not all date nights are created equally, and some people might be more adept at planning dates that promote closeness. Drawing from the self-expansion model and relationship goals literature, we predicted that people higher (vs. lower) in approach relationship goals would be more likely to plan dates that are more exciting and, in turn, experience more self-expansion from the date and increased closeness with the partner. In Study 1, people in intimate relationships planned a date to initiate with their partners and forecasted the expected level of self-expansion and closeness from engaging in the date. In Study 2, a similar design was employed, but we also followed up with participants 1 week later to ask about the experience of engaging in their planned dates (e.g., self-expansion, closeness from the date). Taken together, the results suggest that people with higher (vs. lower) approach relationship goals derive more closeness from their dates, in part, because of their greater aptitude for planning dates that are more exciting and promote self-expansion.

Popular advice given to romantic couples to maintain their connection is to “plan a date night” (e.g., Gottman et al., 2019), yet not all planned dates are equally effective. Although couples might experience temporary boosts in relationship quality from engaging in familiar, comfortable activities such as going to dinner and a movie (Muise et al., 2019), according to the self-expansion model, one way to enhance and sustain closeness in established relationships is to engage in exciting, shared leisure activities that promote a broadening of the mind and a new perspective of the self (i.e., self-expansion; Aron & Aron, 1986, 1996; Aron et al., 2013; Branand et al., 2019). Indeed, engaging in exciting activities with a partner can be self-expanding (Harasymchuk et al., 2020; Muise et al., 2019), and associated with greater satisfaction and closeness (Aron et al., 2000; Graham, 2008; Harasymchuk et al., 2020; Muise et al., 2019).

Some people might be more adept than others, however, at creating and capitalizing on opportunities for self-expansion in their relationships. For instance, people with strong approach relationship goals—those who are motivated to pursue intimacy and growth in relationships (Gable, 2006)—report a greater daily occurrence of exciting, shared activities in their relationships (Harasymchuk et al., 2020). What remains to be understood is whether people high (vs. low) in approach relationship goals are better at creating such opportunities when planning dates as opposed to simply letting them spontaneously occur. Although planning might seem like the antithesis of excitement, pre-arranging these activities might actually ensure they happen and facilitate feelings of excitement. For instance, planning and initiating a day trip to visit a new town with a partner might create an environment for non-routine, spontaneous, and exciting moments to naturally emerge. The goal of this research was to examine whether people higher, relative to lower, in approach relationship goals have a greater aptitude for planning dates that are exciting and, in turn, promote greater self-expansion (i.e., broadening one’s perspective of themselves and the world) and, ultimately, greater closeness with their partner.

## Shared leisure activities in relationships

Maintaining a satisfying relationship extends beyond managing conflict and reducing negative affect (i.e., threat mitigation); it also involves increasing positivity and promoting leisure (i.e., relationship enhancement; Ogolsky et al., 2017). Shared recreation and joint leisure activities are examples of interactive maintenance behaviors that have been identified as important markers of relationship quality (e.g., Busby et al., 1995; Fowers & Olson, 1993). Despite the seemingly inconsequential nature of shared leisure activities and viewing them as a “bonus activity” (Claxton & Perry-Jenkins, 2008, p. 28), growing evidence suggests that shared recreation is important for promoting closeness in established relationships (e.g., Aron et al., 2000; Coulter & Malouff, 2013; Rogge et al., 2013). One important model that explains why certain forms of shared recreation is beneficial to relationships is the self-expansion model.

## Self-expansion in relationships

According to the self-expansion model, people actively seek to expand their sense of self, perspective, and identity (Aron & Aron, 1996, see Aron et al., 2013 for a review). New relationships often provide opportunities for self-expansion because people are gaining new information about their partner and may be incorporating their partner’s attributes into their sense of self (Aron & Aron, 1986; Xu et al., 2016). As partners become increasingly interdependent, they begin to feel that their lives (and self-concepts) are intertwined and closer (Aron & Aron, 1986; Aron et al., 1992). As a result, it is common for relationship satisfaction and love to be high in the early stages of a relationship (Aron et al., 2004). However, over time, as the partner becomes more familiar, there are fewer opportunities to gain new perspectives and have novel experiences (Aron & Aron, 1986, 1996).

Couples can sustain self-expansion over time in ongoing relationships by engaging in exciting shared leisure activities. Excitement in relationships can be defined in a variety of ways (e.g., novelty, arousal, challenge, see Aron et al., 2013) as well as by features such as interest, spontaneity, playfulness, and adventure (Malouff et al., 2012). There is mounting evidence that, despite the potentially risky nature of exciting activities (e.g., fear of embarrassment, departure from security; Bacev-Giles, 2019, see also Aron et al., 2001 for a discussion), engaging in these activities with a partner can promote higher relationship quality (Aron & Aron, 1986, 1996; Aron et al., 2000; Graham, 2008; Harasymchuk et al., 2020; Muise et al., 2019). Researchers have examined the beneficial effects of exciting shared activities in the context of the lab (Aron et al., 2000), by giving homework instructions (Coulter & Malouff, 2013), and by measuring exciting activities as they naturally occur in people’s daily lives (Harasymchuk et al., 2020). Thus, the focus of much of this past work has been on the outcomes of exciting shared activities. However, less is known about the antecedents, such as what occurs when shared activities are planned and initiated, and whether some people are more successful at doing so (i.e., planning exciting dates) than others.

## Approach relationship goals

People differ in terms of their motivational orientation in the context of relationships: some people are motivated by goals aimed at achieving positive outcomes such as intimacy and growth (*approach relationships goals*) and others are motivated by goals aimed at avoiding negative outcomes such as rejection and conflict (*avoidance relationship goals*; Elliot et al., 2006; Gable, 2006; Impett et al., 2010). There is an increasing amount of evidence suggesting that people with higher approach-related motivation have greater relationship quality. For instance, people who score higher on approach-related motivation measures have reported more constructive and creative conflict resolution, more support from the partner (e.g., Winterheld & Simpson, 2011), and have greater responses to positive social events like gratitude and capitalization (Don et al., 2020). As well, people with higher approach relationship goals experience more positive relationship outcomes such as increased relationship satisfaction and closeness (assessed over a 2-week period and as rated by observers; Impett et al., 2010), greater responsiveness toward their romantic partner (Impett et al., 2010), greater sexual desire over a 6-month period (Impett et al., 2008), and more effective (i.e., more satisfying, less reported conflict) forms of sacrifice in relationships (Impett et al., 2014). In contrast, avoidance relationship goals have been associated with decreased relationship satisfaction over time (Impett et al., 2010, 2014), as well as lower observed responsiveness to a partner in a lab interaction (Impett et al., 2010).

Approach relationship goals (Harasymchuk et al., 2020) and other approach motivation-related variables (Mattingly et al., 2012, 2014) have also been linked to self-expansion. For instance, in a daily diary study involving couples, on days when people (or their partners) had higher daily approach relationship goals than typical, they were more likely to engage in an exciting couple activity, which was associated with increased relational self-expansion and, in turn, greater relationship satisfaction (Harasymchuk et al., 2020). As well, Mattingly et al. (2012) found that, across three studies, participants who scored higher on approach motivation-related variables (i.e., motives related to sacrifice, promotion-oriented regulatory focus, behavioral activation system) reported more relational self-expansion (i.e., expansion derived from or in the presence of their partner). In terms of why people with high approach relationship goals might experience more exciting activities (and self-expansion more broadly), one potential reason is that they want to engage in these activities more—at least in the context of relationship initiation (Mattingly et al., 2012). That is, they might be more attuned to opportunities for excitement and might also be more likely to capitalize and engage in them at the first chance. Further, although this idea has not been examined, it is possible that people high in approach relationship goals might be better at creating self-expanding opportunities, that is they might have a greater aptitude (perhaps due to a combination of greater practice and affinity toward such activities). Thus, we know that people with high approach relationship goals experience more exciting, self-expanding shared activities with their partner, but we do not yet fully understand how they arrive at that point (i.e., why they report more exciting shared activities). Our goal is to understand whether approach relationship goals are associated with planning dates and whether people higher in approach relationship goals have a greater ability to plan more exciting dates that in turn, promote self-expansion and increased closeness.

## Overview and hypotheses

**Figure 1.**
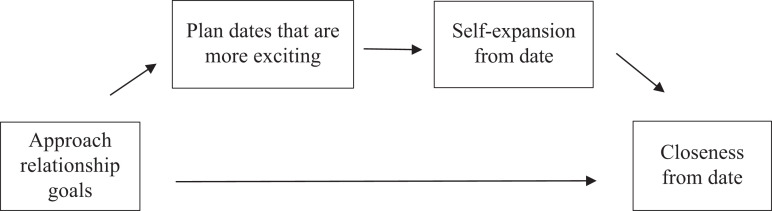
Conceptual model.

The focus of the current research is on planning dates with a romantic partner. We proposed that people with higher (vs. lower) approach relationship goals will plan dates with their partner that are more exciting, and in turn, will report higher self-expansion from engaging in their dates and report greater closeness with their partner from the date (see Figure 1). With regard to avoidance relationship goals, past research has found that individual differences in avoidance relationship goals were not associated with self-expansion experiences (Mattingly et al., 2012). Thus, consistent with past work in this area, we treated avoidance relationship goals as a control variable in our analyses to provide evidence that it is approach goals in particular—rather than relationship motivation more generally—that drives self-expansion and associated outcomes.

There are several potential novel contributions of our research. First, we are investigating an understudied context in the area of self-expansion—that is, the planning or what transpires before the occurrence of an exciting shared activity (vs. the outcomes). Second, we are examining the aptitude of certain people (i.e., high approach relationship goals) to generate, in advance, the types of dates (i.e., more exciting), that have the potential to enhance self-expansion and closeness. Finally, we are focusing on specific date experiences and examining what people forecast to gain from the planned date (Studies 1 and 2) and the outcomes of the planned date experience (Study 2).

To test these hypotheses, we conducted two studies with samples of individuals in intimate relationships. In Study 1, we examined whether people who scored higher (vs. lower) in approach relationship goals planned dates that are more exciting (as rated by themselves and by independent coders) when given the opportunity to plan *any* type of date to initiate with their partner. In addition, as an initial test of the model, we had participants forecast the expected level of self-expansion and closeness from engaging in the date. In Study 2, we aimed to replicate the findings of Study 1 as well as build on them by following up with participants (within 1 week) to ask about the outcome of their dates (e.g., self-expansion, closeness from the date).

## Study 1: Date planning

In Study 1, we examined whether people with higher (vs lower) approach relationship goals generate dates that are more exciting when planning a date to initiate with their partner. In this study, participants could design *any* type of date, *without any instructions* about its qualities (e.g., about it being exciting).^1^ This design differs from past studies that have provided guidelines about the types of exciting activities that couples engaged in outside of the lab (e.g., Coulter & Malouff, 2013; Reissman et al., 1993). Additionally, rather than an “exciting: yes or no” format that has been used in more naturalistic assessments (e.g., Harasymchuk et al., 2020), participants rated the extent of exciting elements in the date. To provide an initial test of the full model, the forecasted levels of self-expansion from the date (i.e., how they expect to grow from the date) and the closeness participants expected to feel with their partner from the date were also assessed. To consider several alternative explanations, we asked about the level of feasibility of the date and participants’ level of relationship satisfaction.

### Method

#### Participants

Participants in romantic relationships for at least 2 months were recruited via Amazon’s Mechanical Turk in exchange for a small monetary compensation (.50 US). Participants had to first pass a CAPTCHA test (to assess for fraudulent responses) before they had a chance to participate. A sample of 193 would allow us to detect small to medium correlations with 80% power. We overrecruited to account for possible exclusions. Seven people were excluded for failing our attention check leaving a sample of (*N* = 251; 66% women). The majority of the participants were either married/common law (48%) or exclusively involved (45%). The remaining 7% were casually dating. The mean relationship length for all participants was approximately 8 years (M_length_ = 92.26 months, *SD =* 108.70 months, ranging from 2 months to 54 years). Participants’ ages ranged from 19 to 87 years old (*M =* 36.58, *SD =* 11.34). The majority of the participants were White (77%), followed by Black (8%), Asian (7%), and other (8%).

#### Procedure and materials

Participants were first asked to complete demographic questions (i.e., gender, age, relationship status, relationship length) and a measure of relationship goals. Next, participants were asked to plan a date to engage in with their partner (“We are asking you to plan a date to engage in with your partner. This date may be anything of your choosing. As well, please indicate when and where the date will take place.”). The “open approach” with minimal instruction was employed because we felt it would be the best way to observe the full range of dates that people high (vs low) in approach relationship goals would generate. Participants were also instructed to make a copy of the date to serve as a reminder. Then, participants assessed the qualities of their planned date they would initiate (e.g., excitement) and completed measures of forecasted self-expansion and closeness from the date.

Participants completed an 8-item relationship goal measure to assess their levels of **approach and avoidance relationship goals** within their romantic relationship (Elliot et al., 2006; Impett et al., 2010). They rated statements relating to how they behave within their current romantic relationship on a scale of (1) “s*trongly disagree*” to (7) “s*trongly agree*” (e.g., “I try to move forward toward growth and development” for *approach relationship goals*, α = .90, and “I try to avoid getting embarrassed, betrayed, or hurt by my romantic partner” for *avoidance relationship goals*, α = .72*)*.

To measure the **exciting qualities of the date** they planned, we used the Four-Factor Romantic Relationship (FFRR) scale, which measures characteristics of romantic relationships on four subscales: excitement, security, care, and stress (Malouff et al., 2012). In the present study, we focused solely on the excitement subscale (9-item measure) and participants rated the extent to which a series of adjectives (e.g., “adventurous,” “playful,” “exciting”) was characteristic of their date on a 5-point scale (1 = *very slightly or not at all* to 5 = *extremely*) which were combined into a composite, α = .85. We also had three trained coders independently rate each of the dates in terms of how exciting they are from the perspective of an outsider on a 5-point scale (1 = *low excitement*, 3 = *moderately exciting*, 5 = *very exciting*). The coders were provided with the excitement scale for the definition of excitement and there was adequate inter-rater reliability (ICC = .66).

We asked participants to rate the extent of **forecasted self-expansion** from the date with three items (“I feel as though I will grow as a person through engaging in this date”; “Engaging in this date will allow me to gain new perspectives”; “I feel that engaging in this activity will give me many opportunities to grow as a person”) on a 6-point scale (1 = *strongly disagree* to 6 = *highly agree*; α = .94. Additionally, **forecasted closeness from the date** was measured using Girme et al.’s (2014) scale of closeness-inducing properties of a date with 4 items: **“**To what extent will engaging in this activity bring you closer together to your partner?”; “To what extent will engaging in this activity provide satisfaction to your relationship?”; “To what extent will the activity make you feel accepted and valued by your partner?”; “To what extent will the activity make you feel close and intimate with your partner?,” rated on a 7-point scale (1 = *will not bring us closer/provide us satisfaction/feel accepted or valued/feel close or intimate at all* to 7 = *will bring us a lot closer/provide us satisfaction/ feel accepted or valued/ feel close or intimate*; α = .90).

To consider several alternative explanations, we asked participants about the **feasibility** of the planned date with 3 items “feasible,” “realistic,” and “likely to engage in the activity” on a 5-point scale (1 = *not at all feasible/realistic/likely* to 5 = *extremely feasible/realistic/likely to engage*, *M* = 4.38, *SD* = .76, α = .83). Additionally, their **relationship satisfaction** was assessed using a 7-item measure (Hendrick, 1988) on a 5-point scale (e.g., 1 = *unsatisfied* to 5 = *extremely satisfied*, *M =* 4.12, *SD =* .77, α = .89).^2^

### Results

People planned a variety of dates including activities involving meals, movies, walks, sports, entertainment (e.g., art, music) and travel. There was sufficient variability in the excitement scores for both the personal ratings and independent coder ratings with the average just above the midpoint of the scale (see Table 1 for descriptives). The excitement ratings from the participants were moderately and positively associated with the independent coder ratings of excitement, *r* = .44, *p* < .001, suggesting that the level of excitement in the planned dates was also recognized by others (see Supplemental for examples of the types of dates the independent coders assessed as low, moderate, and high in terms of excitement).

**Table 1.** Relationship goals and date correlates in studies 1 and 2.

	1	2	3a	3b	4	5	6	7
***Study 1***								
1. Approach goals	—							
2. Avoidance goals	.34***	—						
3. Planned date excitement								
a. Self-rating	.36***	.06	—					
b. Coder-rating	.13^t^	.04	.44***	—				
4. Forecasted self-expansion	.26***	.05	.58***	.29***	—			
5. Forecasted closeness	.49***	.19**	.59***	.25***	.45***	—		
M (SD)	5.81 (1.03)	5.29 (1.10)	3.21 (.81)	2.43 (.84)	4.01 (1.27)	5.60 (1.13)		
***Study 2*** *(replication)*								
1. Approach goals	—							
2. Avoidance goals	.43***	—						
3. Planned date excitement								
a. Self-rating	.32***	.08	—					
b. Coder-rating	.10	−.005	.38***	—				
4. Forecasted self-expansion	.18**	.01	.62***	.30***	—			
5. Forecasted Closeness	.56***	.14*	.63***	.18**	.50***	—		
*Study 2 (follow-up)*								
6. Self-expansion	.26***	.05	.47***	.23**	.52***	.30***	—	
7. Closeness	.49***	.19**	.43***	.09	.25**	.45***	.55***	—
M (SD)	6.03 (.89)	5.45 (1.14)	3.27 (.86)	2.27 (.98)	4.02 (1.33)	5.72 (1.16)	3.80 (1.33)	5.46 (1.26)

Note. *** = *p* < .001; * = *p* < .05, ^t^ = *p* < .10. Goals and closeness from the date (including forecasted) were rated on a 7-point scale, excitement (self and independent coder ratings) on a 5-point scale, and self-expansion (including forecasted) on a 6-point scale.

Do people who score higher (vs. lower) on approach relationship goals generate date ideas that are more exciting when asked to plan a date to initiate with their partner? To assess our hypotheses, we treated approach and avoidance relationship goals as simultaneous predictors with excitement ratings from the self and coders, as well as forecasted self-expansion and closeness from the date as the outcomes (in four separate analyses; see Table 1 for correlations). These results revealed that people high in approach relationship goals planned dates that were more exciting (self-rated), *b* = .29, *SE* = .05, CI [.19 to .40], β = .38, *p* < .001, and this pattern of findings was replicated with the ratings of three outside coders, albeit, the association only reached marginal significance, *b* = .10, *SE* = .06, CI [−.02 to .21], β = .12, *p* = .09. In terms of forecasted outcomes of the date, people who scored higher on approach relationship goals expected more self-expansion from the date, *b* = .32, *SE* = .09, CI [.15 to .49], β = .26, *p* < .001, as well as more closeness from the date, *b* = .54, *SE* = .07, CI [.40 to .67], β = .49*, p* < .001. Avoidance relationship goals did not significantly predict any of the variables in the model (*ps* > .22). The variances for the reported models were *R* ^2^ = .13, *R* ^2^ = .02, *R* ^2^ = .06, and *R* ^2^ = .25 (respectively).

An initial test of our model focusing on the self-ratings of excitement was conducted (the independent coders’ ratings of excitement did not meet the statistical threshold). We employed a serial mediation model, adapted from Harasymchuk et al. (2020), to assess our prediction that people high in approach relationship goals will forecast more self-expansion from the date and expect to experience more closeness with their partner because they have a greater aptitude for planning dates that are more exciting. Our focal variables were approach relationship goals (predictor), excitement level of the planned date (first mediator), forecasted self-expansion from the date (second mediator), and forecasted closeness from the date (outcome), while controlling for avoidance relationship goals. Indeed, these analyses revealed that people high in approach relationship goals planned dates that were more exciting (*b =* .29, *SE* = .05, CI [.19 to .40], *p <* .001). Planning a date that was more exciting was associated with more forecasted self-expansion (*b =* .88, *SE* = .10, CI [.69 to 1.08], *p <* .001), and, in turn, greater forecasted self-expansion was associated with more forecasted closeness (*b =* .16, *SE* = .05, CI [.05 to .26], *p =* .004). The indirect effect from approach relationship goals to the closeness of the date, through planning exciting dates and experienced self-expansion was significant (*b =* .04, *SE* = .02, CI [.01 to .08]). The variance for the model was *R* ^2^ = .13.

#### Considering alternative explanations and generalizability

We also wanted to assess whether the date people planned was something they thought was possible (i.e., not just an unlikely, aspirational date). People high in approach relationship goals planned more feasible dates (*r* = .25, *p* < .001) and the pattern of associations between approach relationship goals and excitement of the date remained consistent when controlling for the feasibility of the date (*b* = .29, *p* < .001). Further, a similar pattern was found when controlling for the level of satisfaction in the relationship (*b* = .29, *p* < .001). Additionally, we explored the role of relationship length and age (i.e., as moderators of approach relationship goals in our regression analyses) and gender differences (assessed the mean differences between men and women for the variables in the model) and found no significant interactions for relationship length and age, nor mean-level gender differences (respectively) for any of the variables.

## Study 2: Date planning and follow-up

In Study 2, we sought to replicate the findings from Study 1 and expand upon them by following up with participants to assess the actual (as opposed to just the expected) outcomes of the dates assessed 1 week later. Even though in Study 1 our participants rated the dates as being relatively feasible and realistic to initiate with their partner, we did not have evidence that they followed through with actually going on the dates. In Study 2, we had people plan a date to initiate with their partner (like Study 1) and asked them to engage in the date with their partner in the coming week. We then followed up with participants 1 week later to ask about their experience of going on the date (e.g., self-expansion and closeness from the date).

### Method

#### Participants

Participants in romantic relationships (*N* = 269) were recruited via Amazon’s Mechanical Turk in exchange for monetary compensation (.50 US for Part 1; 2.25 US for Part 2). To be eligible for participation, participants had to (a) currently be in a romantic relationship, (b) be in a geographically close relationship with their partner (i.e., no long distance), and (c) report that they would see their partner over the following 6 days (as well as pass the initial CAPTCHA to assess for fraudulent responses). A sample of 193 would allow us to detect small to medium correlations with 80% power. We overrecruited to account for eligibility exclusions. Twenty-one participants failed our attention check at Time 1 and were excluded from the analyses leaving a sample of *N* = 248 (64% women). The majority of the participants were either married/common law (55%) or exclusively involved (38%) (the remaining 8% were casually dating). The mean relationship length for all participants was approximately 8 years (*M*_length_ = 100.36 months, *SD =* 103.36 months, ranging from 1 month to 50 years). Participants’ ages ranged from 19 to 68 years old (*M =* 36.49, *SD =* 10.18). The majority of the participants were White (86%), followed by Black (8%), Asian (3%), and other (3%). The sample was relatively satisfied in their relationship, (*M* = 4.19, *SD* =.74) on a 5-point scale.

Of the original 248 participants, 168 (68%) completed the follow-up questionnaire; however, 18 of those participants did not report engaging in the date they originally planned, leaving a final sample of *n* = 150 participants. Importantly, there were no significant differences for any of the variables in the model (nor demographic variables) for the people who completed both time points versus those that completed only the first time point.

#### Procedure and materials

The first part of the study was nearly identical to the procedure and measures used in Study 1. That is, participants completed a measure of demographics and relationship goals and were then given instructions to plan a date with their partner that they would initiate and participate in with their partner in the upcoming week and rate its qualities. Unlike Study 1, participants were additionally prompted to plan a date that was reasonable to engage in during the next 6 days. All measures reached acceptable levels of reliability: approach relationship goals (α = .89), avoidance relationship goals (α = .77), self-ratings of excitement (α = .88), forecasted self-expansion (α = .96), forecasted closeness from the date (α = .89), feasibility of the date (α = .79), and relationship satisfaction (α = .89; see Table 1 for Study 2 descriptives). As well, the reliability of the three independent coders (same coders as Study 1) for ratings of excitement in the date was good (ICC = .76).

Just under 1 week following the initial session, participants completed an online follow-up questionnaire in which they assessed the outcomes of their date. To assess the outcomes of the date, we used an adapted version from Study 1 and asked participants to rate the extent of **self-expansion** from the date with 3 items (e.g., “I feel as though I have grown as a person through engaging in this date”) on a 6-point scale (1 = *strongly disagree* to 6 = *highly agree*; α = .96). Additionally, **closeness from the date** was measured with Girme et al.’s (2014) 4-item measure adapted from Study 1 (e.g., “To what extent did engaging in this activity bring you closer together to your partner?”) on a 7-point scale (e.g., 1 = *did not bring us closer at all* to 7 = *brought us a lot closer*; α = .91.^3^

### Results

We first replicated the findings from Study 1 with our sample from Time 1 (see Table 1 correlations and descriptives).^4^ Consistent with Study 1, people who scored higher on approach relationship goals planned dates that were more exciting, as rated by themselves, *b* = .34, *SE* = .07, CI [.21 to .46], β = .35, *p* < .001 and by outside observers, albeit the latter was marginally significant, *b* = .13, *SE* = .08, CI [−.02 to .29], β = .12, *p* = .09. As well, people higher in approach relationship goals forecasted more self-expansion from the date, *b* = .32, *SE* = .10, CI [.11 to .52], β = .21, *p* = .003 and more closeness from the date, *b* = .80, *SE* = .08, CI [.65 to .95], β = .61, *p* < .001. Avoidance relationship goals were not significantly associated with any of the variables in the model (*ps* > .23), with the exception of forecasted closeness, *b* = −.12, *SE* = .06, CI [−.24 to −.005], β = −.12, *p* = .04. The variances for the reported models were *R* ^2^ = .10, *R* ^2^ = .01, *R* ^2^ = .04, and *R* ^2^ = .33 (respectively). The serial mediation with forecasted outcomes also replicated (with a similar pattern of associations as Study 1), as the indirect effect was significant (*b =* .05, *SE* = .02, CI [.02 to .09]). The variance for the model was *R* ^2^ = .10. The results were maintained when controlling for the feasibility of the date as well as relationship satisfaction (with exception, not for independent ratings of excitement for feasibility).^5^

Unique to this study, we followed up with participants after we gave them time to engage in the date to assess the outcomes (rather than just forecasted outcomes) of date planning. We employed a serial mediation model and our focal variables were approach relationship goals at Time 1 (predictor), how exciting the planned date was at Time 1 (first mediator), self-expansion from the date at Time 2 (second mediator), and closeness from the date at Time 2 (outcome), while controlling for avoidance relationship goals at Time 1 (see Table 1 for correlations). We conducted the model only for self-ratings of excitement because the independent ratings of excitement were only marginally associated with approach relationship goals.

Consistent with our main hypothesis, people high in approach relationship goals planned dates that were more exciting (*b =* .31, *SE* = .09, CI [.13 to .49], *p <* .001). Planning a date that was more exciting at Time 1 was associated with more self-expansion experienced from the date, measured at Time 2 (*b =* .71, *SE* = .12, CI [.48 to .95], *p <* .001), and, in turn, greater experienced self-expansion was associated with greater closeness from engaging in the date (*b =* .44, *SE* = .07, CI [.30 to .59], *p <* .001). The indirect effect from approach relationship goals to the closeness of the date, through planning exciting dates and experienced self-expansion was significant (*b =* .10, *SE* = .04, CI [.03 to .19]).

#### Considering alternative explanations and generalizability

The findings in the model were maintained when controlling for participants’ relationship satisfaction at Time 1. Controlling for relationship length, age, or gender did not alter the serial mediation model. As well, there were no mean differences between men and women for the outcomes of the date.

## General discussion

Although the benefits of shared self-expanding activities in relationships (e.g., exciting date nights) are well supported (Aron et al., 2000; Graham, 2008; Muise et al., 2019), less attention has been placed on what *leads up* to their occurrence. In this research, we asked people in romantic relationships to plan shared leisure activities (Studies 1 and 2) as well as followed up with them after we gave them time to engage in these activities (Study 2 only). The primary focus of our research was on whether people high in approach relationship goals—those who are focused on promoting positive outcomes in their relationships—have a greater aptitude for planning the types of dates that promote self-expansion and closeness (i.e., dates that are more exciting). Drawing from the self-expansion model and relationship goals literature, we found evidence that people who score higher (vs. lower) on approach relationship goals *generate* date ideas that are more exciting (as rated by themselves and independent raters) when asked to plan dates to initiate with their partner. In addition, they forecasted more self-expansion and closeness with their partner from the dates they planned. In our “plan and follow-up” design in Study 2, we found that people who scored higher (vs. lower) in approach relationship goals reported experiencing more self-expansion and, in turn, increased closeness with the partner from engaging in the date because they planned dates that were more exciting (as rated by the self).

### Promoting closeness by planning to self-expand

One of the novel contributions of our research is that we examined a context in which people can be proactive in promoting self-expansion in their relationship, namely by planning exciting dates with their partner. To date, the self-expansion model has not been examined in the context of how shared activities are planned and initiated. Scholars suggest that it is best to engage in these activities as a preventative measure, rather than waiting until relationship decline sets in (e.g., boredom) to react and initiate these types of activities (i.e., Aron & Aron, 1996; Reissman et al., 1993). Indeed, there is supporting evidence that although people know what they should do when they are bored in their relationships (i.e., engage in exciting activities with their partner), they are not necessarily more likely to do so (Harasymchuk et al., 2017). Although planning might seem contrary to the intention of exciting activities (i.e., to be spontaneous and “in the moment”), it might help to set the stage for an environment in which a couple can have natural, unprompted, and free moments to explore and play (i.e., sorting out the logistics to maximize desired choices). This fits existing research on planned leisure outside the relational domain, in the context of vacations, where planning is viewed as an essential feature that contributes to the success of the trip (Hwang et al., 2019; Stewart & Vogt, 1999).

#### Proclivity to plan for self-expansion: The role of approach relationship goals

When planning dates, some people might be more adept at planning activities that promote self-expansion. In this research, we not only found that people high in approach relationship goals generated dates that they found more exciting, they also generated dates that outsiders (i.e., independent coders) rated as more exciting. Consistent with self-expansion theorizing that it matters more what the person thinks (e.g., see Aron et al., 2013), the effects were stronger for self-ratings than for the independent coder ratings. Indeed, it was only when the data sets were combined and there was greater power to detect the effects that the association between approach goals and the excitement of the date (as rated by independent raters) reached statistical significance (see Footnote 5). The focus of past research and theorizing has been on whether the people in the relationship consider shared activities to be exciting, regardless of how the activities are objectively rated (Reissman et al., 1993). For instance, one couple’s exciting activity, such as attending a play, might be another couple’s version of a pleasant or even boring activity. Thus, although research suggests that a wide variety of activities are considered exciting and that people differ in terms of what they consider to be exciting (Aron et al., 2013), our findings offer some initial insight that the dates that are being generated by people high in approach relationship goals are also objectively rated as more exciting as well. The dates rated as more exciting by independent raters had elements of adventure, of making an active effort to travel a longer distance to explore a new environment. Further, the more exciting dates generally involved multiple activities with different experiences and environments to explore (see Supplemental).

In addition to planning dates rated as more exciting, people higher (vs. lower) in approach relationship goals also forecasted more self-expansion from their dates in both studies. In other words, they planned an activity with their partner that would allow them to self-expand. This is another contribution of our research because, to date, scholars have not examined whether people knowingly engage in activities that promote self-expansion. Our research suggests that people, particularly those high in approach relationship goals, are cognizant about engaging in activities with their partner that allow them to see the world in a new light.

#### Extension of the motivational model of self-expansion to the planning stage

Our results complement Harasymchuk et al.’s (2020) findings that have shown that people higher in approach relationship goals report a *greater occurrence* of daily exciting activities which, in turn, is associated with higher daily relational self-expansion. Importantly, our results provide a valuable extension by examining how people high in approach relationship goals might have arrived at the point of an exciting activity occurring with the partner. With our plan and follow-up design, we found that people high in approach relationship goals that planned and initiated a more exciting activity reported experiencing increased self-expansion when engaging in the date with their partner. Thus, from the outset, people with higher (vs. lower) approach relationship goals are being proactive in selecting dates that help them to self-expand with their partner. As well, in these studies, our focus was on how people rated the outcomes of the date itself (vs. the daily relationship outcomes), providing further support for the idea that it is the shared exciting activity itself that contributes to increased self-expansion.

### Limitations and future research

There were several strengths of this research including examining the self-expansion model in a different context (i.e., planning). In addition, we examined the types of dates people planned and then followed up to examine the outcomes. Despite the strengths, there are several limitations. First, we focused on one person’s perspective of planning, initiating, and engaging in a shared activity, but we do not know about the partner’s experience (i.e., whether they found the activity exciting, self-expanding, and closeness inducing), nor do we know what occurs during the planning when both partners try to negotiate their preferences. In future research, it will be valuable to explore this with samples of couples in the lab. Second, our serial mediation model in Study 2 implies a form of temporal sequence and although assessed at two time points, approach goals and exciting date qualities were measured at the same time point (during the planning stage) and self-expansion and closeness from the date were measured at the same time (i.e., after engaging in the date). Future research could tease apart these associations by examining, for instance, whether people primed with approach relationship goals are more apt to plan exciting activities to establish the causal direction. Third, although we tried to limit the recall time by following up with participants within a week, it is possible that people had to recall a date that they had gone on several days earlier. It will be beneficial in future research to assess how people feel during and immediately following the date. Fourth, based on our study, we do not know whether planning enhances or detracts from self-expansion and closeness because we had all participants plan their dates. Future experimental research could examine whether the act of planning enhances the experience or detracts from it. Additionally, research could explore the boundaries of the benefits of planning (e.g., downsides of over-planning or one partner engaging in most of the planning in the relationship). Fifth, we did not assess and control for the level of income or SES in our studies. Many of the more exciting dates involved greater expenses (e.g., travel). In future research, SES could be assessed and controlled for to examine the types of highly exciting dates people with more limited finances might plan. Sixth, although our focus was on approach relationship goals as a predictor of planning more exciting dates, we acknowledge that there are likely other related individual difference variables (e.g., desire for expansion, sensation-seeking, and openness to experience), that might similarly predict date qualities. Finally, we relied on samples of American participants from Mechanical Turk and the findings might not generalize to samples of people from different countries and to people that do not frequent the Mechanical Turk site.

## Conclusion

Shared recreation is important for promoting closeness in established relationships; however, not all date nights are created equally, and some people might be more adept at planning dates that promote closeness. Our results suggest that people high (vs. low) in approach relationship goals are better at being proactive in terms of generating, planning, and initiating the types of shared recreation (i.e., exciting activities) that broaden the mind and ultimately enhance closeness. In other words, people high in approach relationship goals might have foresight into the types of dates that will enhance their relationship quality; that is, the excitement that they experience is not solely spontaneously occurring. This research contributes to a greater understanding about what promotes effective shared recreation and, more broadly, why some couples flourish.

## Supplemental material

Supplemental Material, sj-docx-1-spr-10.1177_02654075211000436 - Planning date nights that promote closeness: The roles of relationship goals and self-expansion Click here for additional data file. Supplemental Material, sj-docx-1-spr-10.1177_02654075211000436 for Planning date nights that promote closeness: The roles of relationship goals and self-expansion by Cheryl Harasymchuk, Deanna L. Walker, Amy Muise and Emily A. Impett in Journal of Social and Personal Relationships

## Notes

1. Data for Studies 1 and 2 were collected before the COVID-19 pandemic.

2. We also assessed how much they thought their partner would want to engage in the date they planned with a single-item, face-valid measure, “To what extent do you feel that your partner will want to engage in this activity with you? (1 = *not at all*, 4 = *moderate desire to participate*, 7 = *strong desire to participate.*” In both studies, people, on average, planned dates in which they thought their partner would want to engage in, *M* = 6.40 (*SD*= 1.01) in Study 1, *M* = 6.40 (*SD*= 1.06) in Study 2. We found that approach relationship goals and anticipated partner desire to engage in the activity were positively associated, *r* = .31, *p* < .001 for Study 1; *r* = .46, *p* < .001 in Study 2. That is, people high in approach relationship goals were being mindful to select dates that would be enjoyable for their partner.

3. In Study 2, we also asked participants about additional features of the experienced date (see Supplemental) including the level of excitement using an adapted version of the measure from the planning portion of the study (5-point scale where 1 = *not at all exciting* and 5 = *very exciting*). There were no significant differences in the level of planned excitement *M* = 3.20, *SD* = .86 and experienced excitement, *M* = 3.27, *SD* = .83, *t* (149) = −1.15, *p* = .25.

4. Unlike Study 1, people who reported being in their relationship for a longer period of time planned dates that were less exciting, *r* = −.21, *p* = .001, and forecasted less closeness from the date, *r* = −.24, *p* = .001; relationship length was unrelated to forecasted self-expansion *r* = −.10, *p* = .12. When assessed as a moderator of approach relationship goals for each of the regression analyses (for replication and the outcomes), none of the interactions with relationship length were statistically significant, βs < |.0005|, *p*s > .12. As well, like Study 1, there were no mean differences for gender for any of the model variables (for the replication, nor the outcomes, *p*s > .12).

5. Given that we obtained the same pattern of marginally significant findings for the independent coder ratings of excitement in Studies 1 and 2, we explored the analyses by combining both datasets. We found that the association between approach relationship goals and independent coder ratings of excitement reached statistical significance, *r* = .10, *p* = .03. As well, the findings were maintained when controlling for feasibility and relationship satisfaction. Furthermore, when assessing the serial mediation model with independent coder ratings of excitement, we obtained the same statistically significant indirect effect as the serial mediation model with self-ratings, *b* = .01 CI [.002 to .03].
