# Supplementary material for: Planning date nights that promote closeness: The roles of relationship goals and self-expansion
Source: J Soc Pers Relat. 2021 Mar 17;38(5):1692–709. doi: 10.1177/02654075211000436 (PMC8170361; doi:10.1177/02654075211000436)
Supplement: Supplemental Material, sj-docx-1-spr-10.1177_02654075211000436 - Planning date nights that promote closeness: The roles of relationship goals and self-expansion [file sj-docx-1-spr-10.1177_02654075211000436.docx]

Supplemental

Excitement is the commonly used way to describe activities that increase self-expansion (e.g., Aron et al., 2000; Coulter & Malouff, Harasymchuk et al., 2020; Reissman et al., 1993); however, there is some debate about what is most essential in shaping self-expansion (see Aron et al., 2013 for a review). In Study 2, we also explored additional correlates of the dates people reported experiencing in the follow-up (i.e., Time 2) including: a 9-item measure of **excitement** adapted from Time 1 (*M* = 3.27, *SD* = .83, α = .91); 3-item measure of **challenge** (challenging, requires a great deal of skill, does not require much ability (rev), *M* = 1.71, *SD* = .88, α = .82); **novelty** (i.e., novel, different, familiar (rev), *M* = 2.58, *SD* = .89, α = .56), and **creativity** (inventive, imaginative, creative, *M* = 2.53, *SD* = 1.15, α = .92), all on a scale of 1-5, where 5 = very exciting/challenging/novel/creative). Participants also completed a 3-item measure of **arousal** on a scale of 1-7, where 7 = high levels of arousal (i.e., alert, involved, active, *M* = 6.20, *SD* = .87, α = .78). See Table 1 for correlations between the features of the experienced date and the main outcomes of our model—self-expansion and closeness from the date.

**Table 1**

Correlations Between the Outcomes of the Date and the Features of the Experienced Date, Measured at the Follow-up (Study 2)

|  | ^1^ | ^2^ | ^3^ | ^4^ | ^5^ | ^6^ | ^7^ |
| --- | --- | --- | --- | --- | --- | --- | --- |
| ^1^ Self-Expansion from the date | 1 |  |  |  |  |  |  |
| ^2^ Closeness from the date | .56*** | 1 |  |  |  |  |  |
| ^3^ Excitement | .64*** | .68*** | 1 |  |  |  |  |
| ^4^ Challenge | .28** | .04 | .31*** | 1 |  |  |  |
| ^5^ Novelty | .52*** | .28** | .59*** | .35*** | 1 |  |  |
| ^6^ Creativity | .55*** | .35*** | .68*** | .29*** | .62*** | 1 |  |
| ^7^Arousal | .36*** | .56*** | .51*** | .12 | .27** | .27** | 1 |

Note. *** = *p* < .001; ** = *p* < .01

As seen in Table 1, all of the features were correlated with self-expansion from the date; excitement was the most strongly related and challenge and arousal were the most weakly related. As for closeness experienced from the date, excitement and arousal were the most strongly correlated; novelty and creativity were more weakly associated; challenge was not significantly associated. Excitement was strongly correlated with the other features of the date, with exception, it was moderately associated with challenge.

Planned Date Examples as Rated by Independent Coders for the Level of Excitement

|  | **Study 1** | **Study 2** |
| --- | --- | --- |
| **Low** |  |  |
|  | *dinner at […] Restaurant and watch a movie at the Movies […]* | *We will rent and watch a movie together. I'll make popcorn and we'll have a home movie night.* |
|  | *At home, large mug of beer or coffee- Marathon run of […] where I explain to him fan theories and plot lines so he can get caught up and we can watch it together without him falling asleep.* | *We will plan to go for a walk while the children are at school. We will hold hands and stop at the park and talk about life. We will eat lunch there and go home.* |
| **Moderate** |  |  |
|  | *I have planned an ice-skating excursion. We would take the train into […] and go skating at […] park. Then, we'd go to have dinner at a nice restaurant.* | *We would drive to our favorite wineries for tastings and finish up at a restaurant in […] at the end of the day.* |
|  | *I would like to go out for breakfast then go climb the indoor rock wall, have a late lunch or early dinner then come home and have a couple of hours in bed together.* | *I am planning a hike up the mountains and a picnic before we head back to the car. I would also love to take pictures of wildlife and just sit in one spot to soak up the sounds, smells and peace of the woods.* |
| **High** |  |  |
|  | *The activity I am planning is to go sky diving with my partner. We would go during sunset hour so we can see the horizon light up. It would probably take place where there are mountains involved.* | *I would love to go out and have a drink at a bar. Then, go to the horse barn. Ride the horses through the trails as the sun goes down. Then, enjoy a nice dinner at a seafood restaurant.* |
|  | *We would be able to go hiking and camping for a few days. That we would bring our own food. Hopefully it would be by a lake or some other type of body of water. We would sleep in a tent.* | *Take a drive up to […] and take a gondola ride to the top of the mountain. Have a romantic picnic lunch overlooking the valley down below. […] Return to town and take a paddle boat ride at […] pond. Afterwards go have a romantic dinner at the […] Bistro overlooking the […] River as the sun sets. After dinner we would take in a concert or movie.* |

Note. Low = dates that rated between 1-2; Moderate = dates rated between 3-4; High = dates rated 4.5-5. Information that was redacted to avoid identifying information is denoted with […]. Coders were asked to make a global excitement rating based not only on the type of activity, but also on the quantity/variety and the way the activities were described.
